# A Comparison between High and Low Cuff Pressures on Muscle Oxygen Saturation and Recovery Responses Following Blood-Flow Restriction Resistance Exercise

**DOI:** 10.3390/s22239138

**Published:** 2022-11-25

**Authors:** Sandro Bartolomei, Pasquale Montesanto, Ivan Malagoli Lanzoni, Giorgio Gatta, Matteo Cortesi, Silvia Fantozzi

**Affiliations:** 1 Department of Biomedical and Neuromotor Sciences, University of Bologna, 40136 Bologna, Italy; 2 Department for Life Quality Studies, University of Bologna, 40136 Bologna, Italy; 3 Department of Electrical, Electronic and Information Engineering, University of Bologna, 40136 Bologna, Italy

**Keywords:** occlusion, strength training, muscle architecture

## Abstract

The aim of the study was to compare the recovery response and muscle oxygenation of a blood-flow restriction resistance exercise (BFR) session with high [HP: 80% of the arterial occlusion pressure (AOP)] and low cuff pressure (LP: 40% of AOP). Both exercise sessions included 4 sets to failure at the barbell preacher curl exercise. Twelve resistance trained men (27.4 ± 5.0 years; 83.5 ± 11.6 kg; 176.6 ± 7.0 cm) performed each protocol in a counterbalanced, randomized order. Maximal isometric force, muscle morphology and muscle soreness of the biceps brachii muscle were assessed at baseline, 15-min, 60-min and 24-h post each testing session. In addition, muscle oxygen saturation (SmO_2_) was assessed during each training session. A lower number of repetitions (*p* = 0.013) was detected in HP compared to LP. A lower SmO_2_ (*p* < 0.001) was detected in the recovery time between the sets in HP (mean: 47.6 ± 15.7%) compared to LP (mean: 68.9 ± 7.2%). No differences between the two trials (*p* > 0.05) were noted for isometric force, muscle architecture and soreness at any timepoint. Results indicate that, despite a high cuff pressure may induce a more hypoxic condition compared to a lower cuff pressure, recovery responses may not be affected.

## 1. Introduction

Resistance exercise with blood-flow restriction (BFR) represents an effective and safe method to increase muscular strength and size in different populations [1]. Several studies to date have investigated the acute and chronic physiological responses following resistance exercise with BFR. Significant improvements in muscle mass have been reported in both young [2] and old [3] individuals training with BFR at low percentages of their maximum strength. Muscle adaptations following BFR resistance training has been reported to be mainly induced by metabolic stress due to the reduction of the blood flowing to the working muscles [4]. Mechanical tension does not appear to be an essential contributor, since BFR exercises are typically performed at 30% of 1 repetition maximum (1RM). However, muscle damage and swelling may also appear following the hypoxic condition obtained with BFR. BFR-induced local ischaemia may also induce a preferential recruitment of fast twitch fibres [5] and that fibre type is more prone to muscle damage. BFR resistance exercise may generate muscle soreness and changes in muscle thickness that can be detected several hours following the BFR exercise session. Although a review by Loenneke et al. [6] reported that muscle damage may not be a crucial factor when low-load BFR exercises are performed, some authors reported changes in sarcolemmal permeability, indicating muscle damages following this type of exercise in active individuals [7]. In addition, significant losses in isometric force, changes in muscle thickness and muscle soreness were found 48 h following a BFR exercise [8].

A parameter that may possibly influence the acute responses following BFR exercise is represented by the pressure of the inflated cuff applied to reduce the blood flow [9]. Recently, some authors [9] suggested a wide range of pressures from 40 to 80% of the arterial occlusion pressure (AOP). These suggestions are inconsistently applied in the experimental studies and many investigations have used arbitrary pressures [10]. Another discrepancy between the different studies is represented by the use of static or dynamic cuffs. Static cuffs are inflated at the beginning of the set or exercise protocol while dynamic cuffs keep the same pressure during the entire exercise protocol using a dynamic pneumatic system [3,9]. Only the first type of cuff, inflated by a standard sphygmomanometer, however, is used in BFR training performed by athletes and sport enthusiasts. Recently, high cuff pressures have been associated with a preferential fast fibres recruitment and more strength adaptations compared to lower pressures. A study by Mattocks et al. [11] reported greater cardiovascular changes and higher rates of perceived exertion (RPE) when high pressures (up to 90% of AOP), compared to low pressures (up to 10% of AOP), were applied. Consistently, Bell et al. [12] reported a lower number of elbow flexions per set when high pressures (80% of AOP) were used compared to low pressures (40% of AOP). These results are also supported by Dankel et al. [13] who reported higher levels of fatigue but similar electromyographic activation during elbow flexions at 20% of 1RM with a BFR at 80% compared to 40% of AOP.

To the best of our knowledge however, no studies to date have compared low and high static cuff pressures for acute changes and recovery responses following BFR exercise for the upper limbs. Thus, the aim of the present study was to compare a high versus low cuff pressure on acute changes in maximal strength and muscle morphology using a typical gym environment. In addition, this study aimed to investigate the influence of the cuff pressure on the recovery response following a BFR exercise session in resistance-trained men. Since BFR exercise for arm muscles is often performed in the attempt to further stimulate muscle hypertrophy in highly trained individuals, in the present study, only resistance-trained participants were recruited.

It was hypothesized that a high pressure of the cuff may induce a more pronounced hypoxic condition and longer post-workout recovery phases for muscle morphology, maximum isometric force and soreness compared to a lower pressure of the cuff. More insights about the difference in the acute and recovery responses between the two conditions of pressure may give important suggestions to improve the quality of the workout. 

## 2. Materials and Methods

### 2.1. Subjects

Participants were resistance-trained men who had participated in regular resistance training for a minimum of 3 times per week in the last 2 years (mean = 12.6 ± 10.5 years of experience). Participants (n = 12; 27.4 ± 5.0 years; 83.5 ± 11.6 kg; 176.6 ± 7.0 cm) were recruited from university sport science classes and among gym-goers. All subjects were between the age of 18 and 35 years and signed an informed consent document after being informed about the risks and benefits of the study. Exclusion criteria included injuries occurring in the year before the study and the use of performance-enhancing drugs. Subjects were not permitted to use dietary supplementation and reportedly did not consume any androgens or other performance-enhancing drugs. Screening for performance-enhancing drug use and additional supplementation was accomplished through a health questionnaire completed at the recruitment stage. Subjects were asked to abstain from alcohol, caffeine and any other relevant physical activity in the 24 h previous to the study. Participants were also asked to maintain their usual nutritional behaviors and were allowed to eat ad libitum. The study was approved by the local University Review Board.

### 2.2. Experimental Protocol

Twelve resistance-trained men participated in the present investigation, consisting of counterbalanced cross-over research. Participants were requested to report to the laboratory on 5 separate occasions. During the first visit they were assessed for anthropometric measures and for 1 repetition maximum (1RM) on the Scott curl exercise. At least 72 h following the initial visit, participants were randomized into the high- or low-pressure trial. Following each workout, they were assessed at 15 min, 60 min and 24 h for muscle soreness, muscle morphology and performance. Participants were requested to report back to the laboratory at least 10 days following the end of the first trial to perform the opposite workout.

### 2.3. Isometric Force Testing

In the first visit participants were tested for anthropometry (body mass and body height) and 1RM at the Scott curl. The 1RM test for the barbell Scott curl was performed using methods previously described by Bartolomei et al. [14]. Briefly, each participant performed two warm-up sets using a resistance of approximately 40–60% and 60–80% of his perceived maximum, respectively. For each exercise, 3–4 subsequent trials were performed to determine the 1RM. A 3–5 min rest period was provided between each trial. Trials not meeting the exercise criteria (e.g., full range of motion) or where technique was not appropriate, were discarded. A qualitative visual judgement was performed by the investigators at this purpose. At least 72 h following the initial visit, participants performed the first trial (HP or LP).

A bilateral isometric biceps curl test assessment was performed using a custom-built instrumented Scott curl, following the muscle morphology assessment. Participants were firmly secured with adjustable straps to the seat with their elbow at 90° of flexion and with hips and knees at 90° of flexion. Joint angles were measured using a goniometer while the participant was seated and stabilized to the device. Grip width and seat height were measured to reproduce the same position for all testing sessions. A strength gauge (Ergo Tester, Globus Inc., Codognè, Italy) was attached to the bar, with the end of the lever arm and perpendicular to it. All isometric assessments were performed using the same setting and positioning. Participants were asked to pull the bar as hard as possible for 5 s. Each participant performed two isometric biceps curls and a recovery time of 2 min was observed between attempts. For this assessment, peak force was measured and the intraclass coefficient was 0.92 (SEM: 86.5 N).

### 2.4. Ultrasound Measurements

Noninvasive skeletal muscle ultrasound images were collected from the participant’s right side. Before image collection, the anatomical locations of interest were identified using standardized landmarks for the biceps brachii muscle (Bic). The probe was positioned on the surface of the skin without depressing the dermal layer (gain = 50 dB; image depth = 5 cm). Muscle thickness (MT) of biceps brachii was measured on the anterior surfaces at 60% of the upper arm length (the distance from the acromion process of the scapular to the lateral epicondyle of the humerus) [15].

Participants were asked to lie on the examination table for a minimum of 15 min before images were collected. A 12-MHz linear probe scanning head (Mindray MD20, Mindray Bio-Medical Electronics Co., Ltd., Shenzhen, China) was coated with water-soluble transmission gel to optimize spatial resolution and used to collect all ultrasound images. All ultrasound images were taken and analyzed by the same expert technician who performed all the landmark measurements. MT measures were obtained using a longitudinal B-mode image and three consecutive MT images were captured and analyzed for each muscle. For each image, MT was measured with a single perpendicular line from the superficial aponeurosis to the deep aponeurosis. The average of the 3 MT measures was used for statistical analyses. Intraclass correlation coefficients for BicMT were 0.96 (SEM = 0.93 mm).

### 2.5. Blood Flow Restriction Protocols

Both trials included 4 sets to volitional failure at the seated preacher curl (on a Scott bench) performed at 30% of 1RM with 30 s of between-set rest. Cadence of the repetitions was standardized using a metronome (2 s for the eccentric phase and 2 s for the concentric phase). The difference between the two trials consisted in the pressure of the inflated cuff. One trial used a cuff pressure corresponding to 40% of AOP (low pressure: LP) while the other trial used a high pressure corresponding to 80% of AOP (high pressure: HP). These pressures represent the lower and the upper limits of the suggested range of pressure [9,12]. Before each trial, standing AOP was determined using a 5-cm-wide nylon cuff (Occlusion Cuff Elite, Theocclusioncuff LTD, Belfast, Ireland) which was placed on the most proximal portion of the arm. A phonendoscope was placed at the wrist over the radial artery. Then the cuff was slowly inflated using a manual inflator until there was no longer any auscultatory pulse [16]. The lowest cuff pressure that suppresses blood flow was defined as arterial occlusion pressure and was registered. The cuff was immediately deflated and removed from the arm after the AOP measurement.

BFR was applied by placing 5-cm wide nylon cuffs to the proximal portion of both arms [12] and inflating them to either 40% or 80% of AOP. In both trials, the cuff was kept on during the entire exercise protocol. Before each trial, the nylon cuffs were checked to detect any eventual leak of pressure. Participants were not assisted by a spotter to complete additional repetitions following volitional failure, thus, forced repetitions were not performed. All resistance and assessment sessions were supervised by the same qualified investigators. Before each trial, subjects performed a standardized warm-up consisting of 5 min cycling on a cycle ergometer against a light resistance, 10 body weight squats, 10 body weight walking lunges, 10 dynamic walking hamstring stretches, and 10 dynamic walking quadriceps stretches [14]. In addition, each participant performed specific warm-up consisting of 2 sets of 8 repetitions of standing biceps curls with 15 kg and 25 kg with 3 min of rest between the sets. 

### 2.6. Muscle Oxygen Saturation

Real-time concentration of oxyhemoglobin and deoxyhemoglobin was monitored during HP and LP protocols using a NIRS (near-infrared spectroscopy) device (MOXY Muscle Oxygen Monitor, Hutchinson, MO, USA). This device calculates the percentage of saturated hemoglobin in relation to the total amount of hemoglobin in the muscle (SmO_2_ = HbO_2_/tHB) using light from the near-infrared wavelength spectrum (light from about 670 to 810 nm) [17]. The amount of light absorbed was analyzed using a modified Beer–Lambert Law. MOXY was placed on the middle of the muscle belly of the left biceps brachii, 8–12 cm above the elbow fold, and wrapped with a dark band to shield ambient light. Muscle oxygenation was sampled at 4 Hz and downloaded on a personal computer using a specific program (MOXY PC, Fortiori Design LLC, Hutchinson, MN, USA). The SmO_2_ at the end of the last repetition of each set (SetO_2_) and the SmO_2_ in the last second of each 30 s between-set recovery (RestO_2_) were used for further analyses. In addition, the change in SmO_2_ from the beginning (SmO_2_ start) to the end (SetO_2_) of each set (ΔSmO_2_) was calculated using the equation previously reported by Gomez-Carmona et al. [18]:

$${\Delta \mathrm{SmO}}_{2\;}\left(\%\right)=\frac{{\mathrm{SetO}}_{2\;}\times 100}{{\mathrm{SmO}}_{2}\mathrm{start}}-100\left(\left(\frac{{\mathrm{SetO}}_{2\;}\times 100}{{\mathrm{SmO}}_{2}\mathrm{start}}\right)-100\right)\times -1{\;}_{\;}$$

The muscle oxygen resaturation rate (SmO_2_Res, expressed in % s^−1^) following each set was also calculated by the SmO_2_ at the end of each set and the value at the end of the 30 s between-set recovery. 

The maximum SmO_2_ (maxSmO_2_) within the first 3 min following the cuff remotion, immediately after the last set of each protocol, was also registered. 

### 2.7. Muscle Soreness and RPE

Fifteen minutes following the conclusion of each training session, participants responded to the question “How was your workout?” avoiding any contact between them, using a session RPE (sRPE) scale [19]. The scale used was as follows: 0 = very easy, 2 = easy, 3 = moderate, 4 = somewhat hard, 5–6 = hard, 7–9 = very hard, and 10 = maximal. 

In addition, 15 min, 60 min and 24 h following each workout, a 100 mm visual analog scale (VAS) was used to assess muscle soreness [20]. No muscle soreness was recorded as 0 and the worst possible soreness as 100.

### 2.8. Statistics

A Shapiro–Wilk test was used to test the normal distribution of the data. If the assumption of sphericity was violated, a Greenhouse–Geisser correction was applied. Data were analyzed using a two-factor (group × time) analysis of variance (ANOVA) with repeated measures to evaluate the differences between the acute effects of the resistance exercise protocols. In the event of a significant F ratio, groups were compared at each timepoints by paired sample *t* tests with Bonferroni correction.

Average ΔSmO_2_ and Rest O_2_ between the two trials were compared using paired sample *t* tests.

Where appropriate, percent changes were calculated as follows: [(post-exercise mean − pre-exercise mean)/pre-exercise mean] × 100. For effect size, the partial eta-squared statistic was reported and according to Stevens [21], 0.01, 0.06, and 0.14 represents small, medium, and large effect sizes, respectively. All data were analyzed using SPSS 20 for Windows (SPSS Inc., Chicago, IL, USA) and are reported in the text as mean ± SD. Significance level was set for *p* ≤ 0.05.

## 3. Results

### 3.1. Number of Repetitions and Muscle Oxygen Saturation

**Figure 1:**
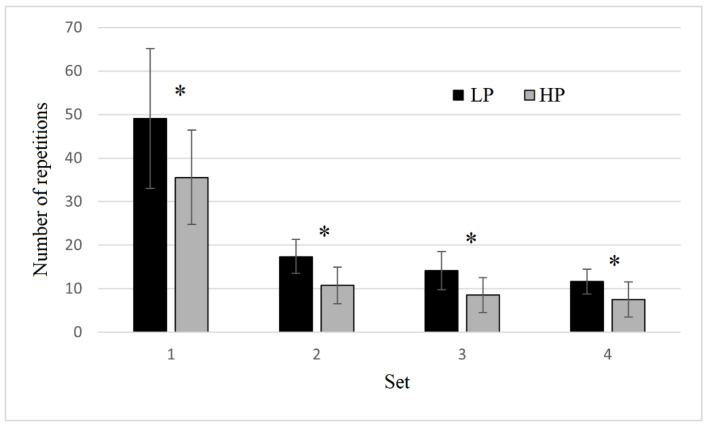
Number of repetitions to failure performed in the four sets provided in both high pressure (HP) and low pressure (LP) trials. * indicates a significant (*p* ≤ 0.05) difference between HP and LP.

The average number of repetitions performed in each set of HP and LP are shown in Figure 1. A significant trial x set interaction (F = 7.552; *p* = 0.013; η^2^ = 0.407) was detected for the number of repetitions. The number of repetitions was lower in HP compared to LP in the first set (−11.1 reps; *p* = 0.006), second set (−5.3 reps; *p* = 0.006), third set (−4.0 reps; *p* = 0.031) and fourth set (−2.9 reps; *p* = 0.044).

**Figure 2:**
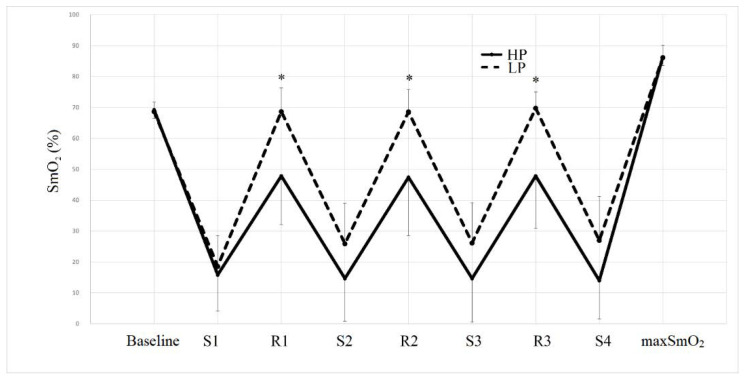
Muscle oxygen saturation (SmO_2_) at baseline, at the end of the first set (S1), at the end of the first inter-set recovery time (R1), at the end of the second set (S2), at the end of the second inter-set recovery time (R2), at the end of the third set (S3), at the end of the third inter-set recovery time (R3), at the end of the fourth set (S4) and within 3 min following the last repetition of the last set (maxSmO_2_). * indicates a significant (*p* ≤ 0.05) difference between HP and LP. HP = high pressure; LP = low pressure.

The pattern of muscle oxygenation during each trail can be observed in Figure 2. A significant trial x time interaction was detected for RestO_2_ (F = 19.750; *p* < 0.001; η^2^ = 0.642). SmO_2_ was significantly lower in HP compared to LP in the recovery time following the first (S1: −30.3%; *p* < 0.001); the second (S2: −30.9%; *p* = 0.001); the third (S3: −31.4%; *p* = 0.001). The average RestO_2_ was 47.6 ± 15.7% and 68.9 ± 7.2% in HP and LP, respectively. This parameter however was not significantly different between HP and LP before the first set (Baseline: *p* = 0.614) and 3 min after the last set (max SmO_2_: *p* = 0.874).

No significant trial x time interactions were noted for SetO_2_ (F = 3.414; *p* ≤ 0.073; η^2^ = 0.237).

Significant differences between the trials were detected for the average SmO_2_Res (*p* = 0.048; CI: 0.862; 0.581), while no differences were found for the average ΔSmO_2_ (*p* = 0.862; CI: −30.68; 35.39). 

### 3.2. Muscle Morphology, Muscle Soreness and RPE Assessments

No significant trial x time interaction was identified for the BicMT (F = 0.284; *p* = 0.742; η^2^ = 0.045). A significant main effect of time was detected for this parameter (F = 12.733; *p* = 0.003; η^2^ = 0.680). This parameter was significantly elevated (*p* < 0.001) with respect to baseline, following both trials at 15P, 60P and 24hP.

**Figure 3:**
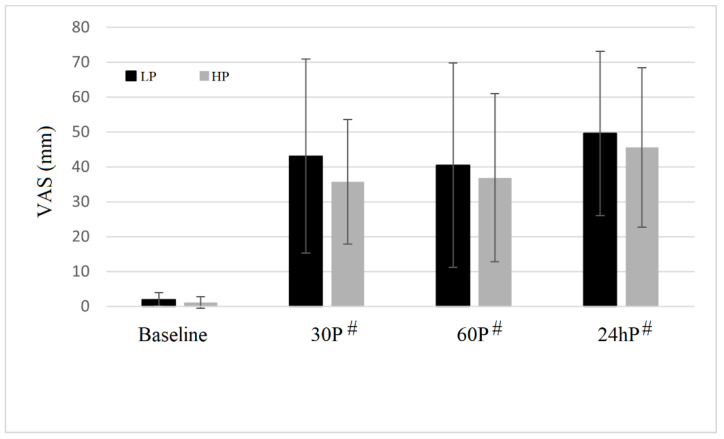
Muscle soreness (VAS = visual analog scale) of biceps brachii muscle at baseline, 30 min (30P), 60 min (60P) and 24 h (24hP) following both high pressure (HP) and low pressure (LP) protocols. ^#^ indicates a significantly difference from baseline.

The results of the VAS for soreness can be observed in Figure 3. No significant trial × time interactions (F = 0.068; *p* = 0.936; η^2^ = 0.001) were observed for soreness intensity. A significant main effect of time was detected for this parameter (F = 13.485; *p* = 0.003; η^2^ = 0.689). Following both trials, VAS was more elevated than baseline (*p* < 0.01) at 15P, 60P and 24hP.

sRPE value was significantly higher in HP (8.1 ± 1.7 a.u.) compared to LP (6.3 ± 2.4 a.u.) (*p* = 0.029; CI: −3.329; −0.216).

### 3.3. Isometric Strength Assessments

All results for isometric bicep curls at the different timepoints in both HP and LP trials are reported in Table 1. No significant trial x time interactions were identified for this assessment (F = 1.250; *p* = 0.320; η^2^ = 0.172). A significant main effect of time was detected for this parameter (F = 3.582; *p* = 0.034; η^2^ = 0.374). In both HP and LP, this parameter was significantly reduced from baseline at 15P (*p* = 0.02) and 60P (*p* = 0.05) only.

## 4. Discussion

The present investigation studied the effects of different cuff pressures (40 and 80% of the arterial occlusion pressure: AOP) on muscle oxygenation (SmO_2_), performance, muscle architecture and muscle soreness following a BFR exercise protocol for the elbow flexor muscles. Results of this study partially confirmed the research hypothesis and showed that a high cuff pressure (HP) reduced muscle oxygenation at the end of the recovery time between sets (RestO_2_) as well as the rate of reoxygenation (SmO_2_Res) following each set, compared to a low cuff pressure (LP). The enhanced hypoxic condition registered in HP compared to LP likely determined a reduction in the number of repetitions performed in each set and elevated RPEs. 

BFR resistance exercise has been associated with acute reductions of maximal voluntary force [22], induced by both central and peripheral fatigue [23]. Moreover, some studies showed that muscle fatigue was more pronounced during BFR compared to regular resistance exercise [24]. Peripheral fatigue induced by BFR has been explained by the accumulation of metabolites (e.g., Pi) and impaired Ca^+^ release by the sarcoplasmic reticulum [25]. In the present study, significantly higher levels of perceived exertion were registered in the HP compared to the LP trial (8.1 and 6.3 for HP and LP, respectively). As previously reported by Bell et al. [12] the highest levels of discomfort are registered when BFR is performed with low loads and high cuff pressures. However, low-load resistance exercises alone lead to greater levels of discomfort compared to heavy-loads resistance exercises. The onset of fatigue and discomfort indeed plays a role in the number of repetitions performed until volitional failure. Interestingly enough, BFR with high cuff pressures is characterized by a significant reduction in the number of repetitions compared to both BFR at low pressures and regular resistance exercise. Thus, high pressure BFR may mainly accelerate the onset of fatigue and discomfort that regularly occurs when sets to failure are performed with light loads [26].

Although similar levels of muscle oxygen saturation (SmO_2_) were registered at the end of each set (SetO_2_), in both HP and LP, lower levels of SmO_2_ have been measured during the between-set recovery in HP compared to LP (RestO_2_). High cuff pressure indeed induced a more hypoxic condition and a lower muscle reoxygenation rate (SmO_2_Res) in this phase, compared to LP. The present study however was not able to detect if the differences between the two protocols in RestO_2_ and SmO_2_Res were due to a reduced arterial inflow or to an accumulation of deoxygenated blood in the venous compartment as a consequence of venous occlusion. Both components indeed may be influenced by the cuff pressure [9], especially during the rest time between the sets. Previous studies indicated a SmO_2_ between 80 and 85% [27] during the rest time between the sets of an upper-body resistance exercise protocol. In the present investigation, SetO_2_ was between 45% and 50% in HP and between 65% and 70% in LP. Recovery is commonly characterized by a hyperemic supra exercise oxygen delivery in the attempt to pay back an oxygen deficit accumulated during the set [28]. Our results confirmed those of Ganesan et al. [26], indicating that BFR reduces SmO_2_ mostly in this phase. In particular, a high cuff pressure reduced the SmO_2_ during the rest time between the sets to a greater extent compared to a lower cuff pressure. Contrarywise, the muscle oxygenation at the end of each set (SetO_2_) was similar compared to a regular resistance exercise set to volitional failure performed with light loads (between 0 and 10%) [27]. 

In the present study, no differences between the two trials were registered in the recovery response following HP and LP. Similar post-workout changes were detected in HP and LP for maximum isometric force, muscle soreness and muscle architecture of the elbow flexor muscles. Maximum isometric force was reduced up to 60 min following both HP and LP trials, without differences between the trials. 

Muscle thickness and muscle soreness were significantly altered 24 h following both HP and LP protocols. This is consistent with previous studies that reported similar acute increases in muscle size and muscle soreness following BFR protocols [8]. These indirect measurements of muscle inflammation may indicate that some muscle damages may occur following both HP and LP. Some authors [8] suggested that the mechanisms of muscle soreness following BFR may be different than those associated with regular resistance exercise. Muscle soreness registered in BFR protocols may be caused by ischaemia-reperfusion injury, leading to large increases in reactive oxygen species. In the present study, muscle oxygenation following the cuff removal was not different in HP and LP, indicating that a similar reperfusion process occurred in the two trials. The similar pattern of muscle soreness, maximal strength and architecture between the two trials also confirms that cuff pressure may not be able to influence the post-exercise recovery response. In addition, the levels of muscle soreness detected in the present study were similar compared to those measured following high-volume regular resistance exercises for the upper [29] and the lower body muscles [30].

A possible limitation of the present study is represented by the use of manual inflatable cuffs instead of an automated tourniquet system [31]. The cuff pressure indeed tends to be more elevated during the concentric phase with respect to relaxation. Another possible limitation is the measurement of the AOP value using a phonendoscope placed at the wrist over the radial artery. Despite some authors recently using this method [32], other determined the suppression of the distal pulse using a doppler probe. Both solutions were adopted by the authors to increase the ecological validity of the study. This condition indeed represents the typical gym setting BFR resistance training. Thus, an important novelty of this study is that the different cuff pressures were compared in a real gym setting condition and using resistance trained individuals.

In conclusion, results of the present study indicate that, despite a high cuff pressure potentially inducing a more hypoxic condition and a lower number of repetitions compared to a lower cuff pressure, similar recovery responses may be registered following BFR protocols with different cuff pressures in trained men.

## Figures and Tables

**Table 1:** Data of the maximum isometric force of the elbow flexors measured at the different timepoints [baseline, 30 min (30P), 60 min (60P) and 24 h (24hP)] in both HP (high pressure) and LP (low pressure) trial. ^#^ indicates a significant difference from baseline.

Timepoint	HP	LP
Baseline	569.0 ± 69.4	569.0 ± 69.4
15P ^#^	528.1 ± 48.3	526.2 ± 39.3
60P ^#^	520.6 ± 82.1	498.6 ± 49.1
24hP	558.0 ± 64.1	527.2 ± 66.0

## Data Availability

Not applicable.
